# Inferring phylogenetic structure, taxa hybridization, and divergence times within rock voles of subgenus *Aschizomys* (Cricetidae: *Alticola*) using quaddRAD sequencing and a *cytb* dataset

**DOI:** 10.1002/ece3.10742

**Published:** 2023-12-13

**Authors:** Ivan A. Dvoyashov, Semyon Yu. Bodrov, Nikolai V. Mamaev, Elena S. Glagoleva, Natalia I. Abramson

**Affiliations:** ^1^ Zoological Institute Russian Academy of Sciences Saint Petersburg Russia; ^2^ Institute for Biological Problems of Cryolithozone Yakutsk Russia; ^3^ Faculty of Biology Lomonosov Moscow State University Moscow Russia

**Keywords:** *Alticola*, hybridization, introgression, phylogeny, quaddRAD‐seq, Siberia

## Abstract

The subgenus *Aschizomys* belongs to the genus *Alticola* (Central Asian mountain vole) and consists of two species: *Alticola macrotis* and *Alticola lemminus.* Phylogenetic relationships within the subgenus *Aschizomys* remain obscure due to limited sampling, an insufficient number of molecular markers used in phylogenetic studies, and paraphyly observed on mitochondrial trees. In this work, to infer reliable phylogenetic relationships and evaluate putative scenarios of ancient hybridization within the subgenus, we applied double‐digest restriction site‐associated DNA paired‐end (quaddRAD) sequencing to 20 DNA samples (20 individuals), including five species of the genus *Alticola*, and dated the divergence of cytochrome b (*cytb*) lineages within *Aschizomys* using a “second calibration” approach. We showed monophyly of the two species on the basis of thousands of nuclear loci and demonstrated traces of introgression also in the nuclear genome. Observed paraphyly in *cytb* could be explained by an introgression event rather than incomplete lineage sorting. This explanation was confirmed by an analysis of the *cytb* divergence time. Overall, our results support the hypothesis of extensive migration of the *Aschizomys* species during the Late Pleistocene, with this migration leading to population divergence and introgression. We expect our article to become a starting point for a series of rigorous studies on the population history of the genus *Alticola* as a whole.

## Graphical abstract

Our case study considers the rock vole, genus Alticola, which remains one of the insufficiently studied genera of voles owing to its hard‐to‐access habitats and low abundance. Phylogenetic structure of the genus is complicated by few cases of mito‐nuclear discordance. In our paper, we focused on one of such cases within the taxon of rock voles, namely the subgenus Aschizomys. The species of this subgenus colonized the northernmost areas within the vast range of the genus. We present here the first genome‐wide study on this group aimed at understanding the causes of the mito‐nuclear discordance and at reconstructing the most likely evolutionary history, phylogeny, and the taxonomy of the subgenus.

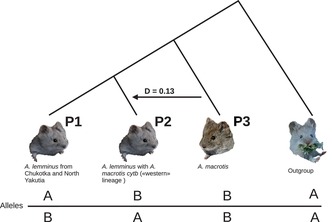

## 1 INTRODUCTION

Paraphyly of a taxonomic group according to some marker is a well‐described issue that arises especially often during comparisons of mitochondrial and nuclear markers (mito‐nuclear discordance; Funk & Omland, 2003; McKay & Zink, 2010; Toews & Brelsford, 2012). When researchers propose the existence of a “species tree,” that is, a tree that reflects actual divergence events between groups, the researchers can expect either a nuclear‐DNA marker or a mitochondrial‐DNA marker to represent this tree. Thus, one can refer to the other marker as discordant, but actually it is unknown which one is discordant until we have the “species tree.” One of the processes leading to mito‐nuclear discordance is incomplete lineage sorting (ILS) on any marker under consideration. Because the mitochondrial genome is haploid and inherited uniparentally—and therefore has an effective population size four times smaller—it will complete the process of lineage sorting faster than the nuclear genome will. Consequently, incomplete sorting is less of a concern for mitochondrial than for nuclear loci, all other things being equal (Funk & Omland, 2003). Nonetheless, there are some authors reporting mitochondrial‐genome ILS leading to mitochondrial tree/species tree discordance (Wang et al., 2018). Another possible reason for mito‐nuclear discordance is the introgression of a discordant marker. An important difference between the two scenarios is that ILS is often considered a stochastic process (Pamilo & Nei, 1988). Therefore, if a discordant marker follows a geographic pattern, then this incongruence is likely to be the result of introgression (Toews & Brelsford, 2012). Moreover, if a “species tree” is known, then the hypothesis of introgression of the discordant marker can be tested by analyzing divergence times of its lineages, as proposed by Joly et al. (2009).

In the last decade, the development of high‐throughput sequencing techniques together with their increasing affordability allowed researchers to analyze thousands of unlinked molecular markers, which dramatically improve the accuracy of phylogenetic reconstruction. This advancement gives investigators more confidence in the “species tree” that they have proposed based on an estimated phylogenetic tree. Moreover, the larger dataset improves the detection of patterns of minor phylogenetic histories: introgression and ILS (Baird et al., 2008; Baum, 2007). Double‐digest restriction site‐associated DNA paired‐end sequencing (quaddRAD‐seq) is one such technique (Franchini et al., 2017). In a number of studies, via this approach (or a similar RAD‐seq approach; Lecaudey et al., 2018; Nash et al., 2018; Sadanandan et al., 2020), the presence of reticulate evolutionary patterns, particularly introgression, has been convincingly shown in closely related species. Moreover, it has become more or less clear that evolution with a reticulate process is not an exception as traditionally thought but rather the rule and involves hybridization and introgression: the primary drivers of mito‐nuclear discordance (Gontier, 2015).

Central Asian mountain voles (i.e., rock voles) of the genus *Alticola* Blanford, 1881, are small rodents inhabiting mountains and lowland uplands up to 6100 m above sea level in the absence of a forest zone. Their preferred habitats are rocky outcrops, talus, or loose vegetation on gravel and pebble surfaces (Gromov & Polyakov, 1997; Musser & Carleton, 2005). The genus occurs in mountain ranges from the Chukotka Peninsula in the northeast to the Pamir–Alai in the west and to Tibet and the Himalayas in the south (Lebedev et al., 2007; Musser & Carleton, 2005). The genus is assigned to the tribe Clethrionomyini (Arvicolinae, Cricetidae, Rodentia) and forms a sister clade with the genus of red‐backed voles *Clethrionomys* Tilesius, 1850. As suggested by paleontological evidence, the two genera diverged 2.0–2.6 million years ago (Alexeeva, 1998; Young, 1934). Twelve species are currently recognized within the *Alticola* genus (Musser & Carleton, 2005). They were initially assigned to three subgenera on the basis of skull morphology and habitual features (Gromov & Polyakov, 1997): *Alticola* Blanford, 1881; *Aschizomys* Miller, 1889; and *Platycranius* Kastschenko, 1901. As later molecular studies have shown, *Platycranius* belongs to the *Alticola* lineage, and thus only two subgenera—*Alticola* and *Aschizomys* (Bodrov et al., 2016; Lebedev et al., 2007)—may be considered valid. The latter consists of two species: *Alticola lemminus* Miller, 1898 and *A. macrotis* Radde, 1862 (Musser & Carleton, 2005), although some authors (Gromov & Polyakov, 1997) suggested that *A. macrotis* is a subspecies of *A. lemminus*. Meanwhile, Bykova et al. (1978) proposed that some populations of *A. lemminus* could be regarded as a separate species based on morphological and karyological characteristics. In the genus, *A. lemminus* is the only species that inhabits mountains of northeastern Siberia from the Chukotka Peninsula to Baikal Lake. *A. macrotis* occurs in the Altai‐Sayan Mountains and according to unconfirmed reports may inhabit mountain ridges of southeastern Transbaikal (Sokhondo, Daursky, Cherskogo, and Yablonovy).

The genus *Alticola* is interesting because of several cases of mito‐nuclear discordance. In previous papers of Kohli et al. (2014) and Lebedev et al. (2007), paraphyly of the genus has been demonstrated on a mitochondrial tree: according to cytochrome b (*cytb*) data, representatives of the subgenus *Aschizomys* are more closely related to the genus *Clethrionomys* than to other *Alticola* taxa. This finding has started the debate on the taxonomic position of the subgenus *Aschizomys* (Lebedev et al., 2007). Meanwhile, the trees built on individual nuclear markers showed monophyly of the genus *Alticola* (Bodrov et al., 2016). Leaving this question for future studies, in this paper, we focused on one more case of mito‐nuclear discordance in this group, namely within the subgenus *Aschizomys* proper. Recent molecular studies (Bodrov et al., 2020; Kohli et al., 2014) indicate the monophyly of the two species of *Aschizomys* in phylogenetic trees based on a few nuclear markers, while *cytb* data show that one of *A. lemminus* populations is placed within the *A. macrotis* clade.

Our study aimed at obtaining inferences concerning mito‐nuclear discordance and determining events of introgression in the subgenus *Aschizomys*. To this end, a genome‐wide single‐nucleotide polymorphism (SNP) dataset was generated through quaddRAD‐seq to (1) infer a nuclear DNA‐based phylogeny of the subgenus and compare it to an existing mitochondrial‐DNA–based topology and (2) test whether the nuclear genome of *Aschizomys* species carries traces of interspecies introgression that could lead to the observed mito‐nuclear discordance. We used the obtained results to infer historical introgression by means of both the genome‐wide SNP data and mitochondrial data. Finally, we discuss all the findings in the context of the history of the subgenus *Aschizomys*.

## 2 MATERIALS AND METHODS

**FIGURE 1:**
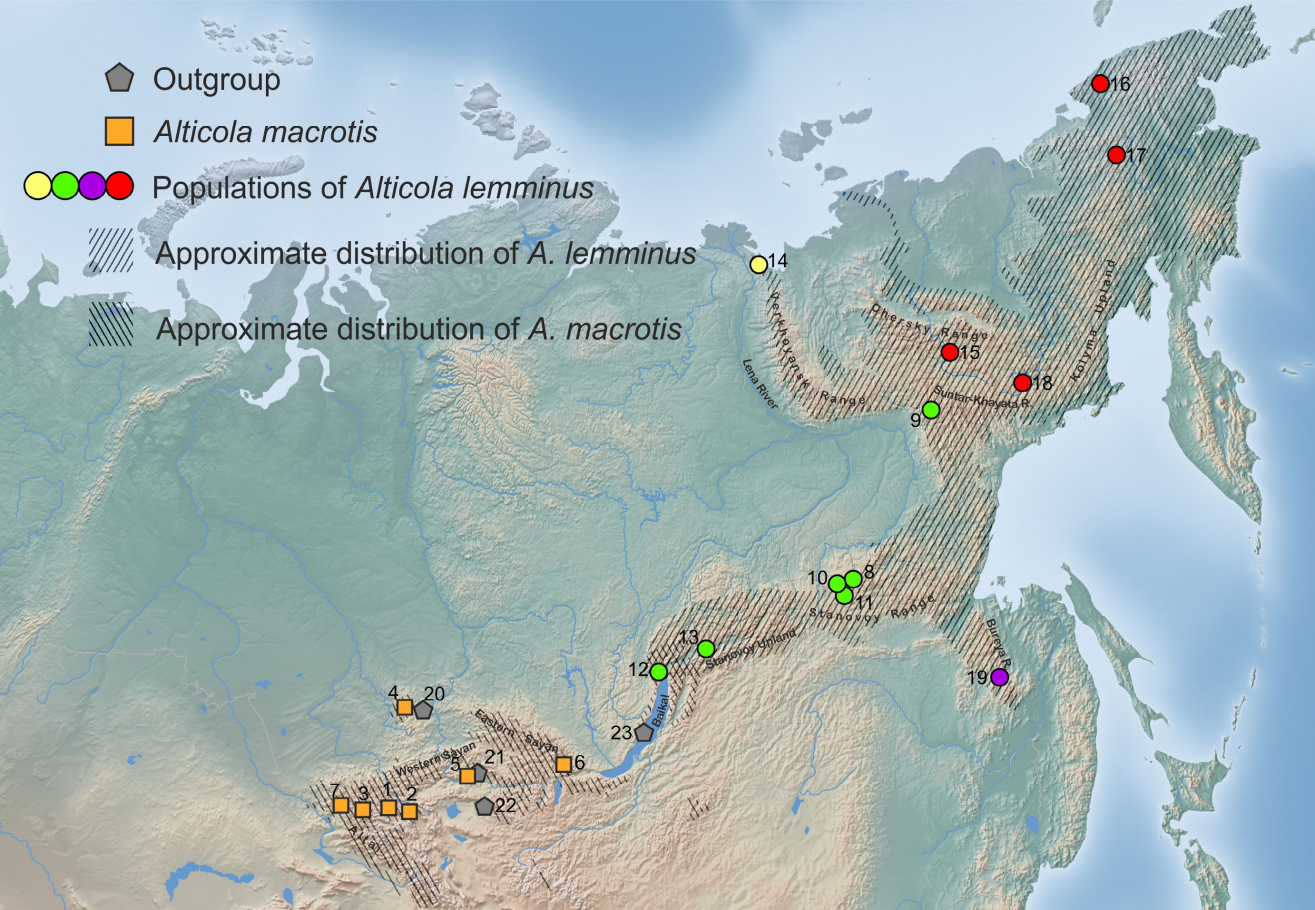
Sampling sites used in the study. Orange squares: *Alticola macrotis* individuals (i.e., DNA samples) All circles indicate different groups of *A. lemminus* individuals: the green color denotes sampling sites of *A. lemminus* that clustered with *A. macrotis* on the *cytb* tree (“western” lineage), the red color means *A. lemminus* individuals from East Yakutia and Chukotka, the yellow color indicates individuals from North Yakutia, and purple indicates individuals from the Bureya mountain range. Gray polygons designate representatives of the outgroup: *A. tuvinicus*, *A. semicanus*, and *A. olchonensis* (subgenus *Alticola*). Hatched areas indicate distribution ranges of *A. macrotis* and *A. lemminus* according to the IUCN Red List of Threatened Species database with changes. Site ID numbers correspond to those in Table 1.

DNA samples analyzed in this work are listed in Table 1, and sampling sites are presented in Figure 1. The cytb dataset consisted of 40 sequences. Twenty seven sequences were downloaded from the NCBI database, three were obtained within this study with PCR amplification and Sanger sequencing (see Bodrov et al., 2020 for details). The remaining 10 were taken from genome‐wide sequencing data available on the NCBI (Table 1).

**TABLE 1:** Materials used in the study.

Taxon	Tissue ID	GenBank accession no. of cytb	quaddRAD‐seq	Site ID	Locality	Northern latitude, °	Eastern longitude, °	Source of cytb
*A. macrotis*	30	MK341117	–	1	Altai Republic, Kurayski Range	50.329	87.773	Bodrov et al. (2020)
–	4116	MK328034	–	2	Altai Republic, Bulyukem River	50.179	89.279	Bodrov et al. (2020)
–	4117	MK341095	Yes	2	Altai Republic, Bulyukem River	50.179	89.279	Bodrov et al. (2020)
–	–	DQ845196	–	3	Altai, Ust‐Koksinsky District	50.07	85.43	Lebedev et al. (2007)
–	5535	OQ992608	–	4	Republic of Khakassia, Ordzhonikidzevsky District	54.612	88.634	SAMN35742100
–	5536	OQ992610	–	4	Republic of Khakassia, Ordzhonikidzevsky District	54.612	88.634	SAMN35742101
–	5537	OQ992609	–	4	Republic of Khakassia, Ordzhonikidzevsky District	54.612	88.634	SAMN35742102
–	5714	OR651409	Yes	4	Republic of Khakassia, Ordzhonikidzevsky District	54.612	88.634	Current study
–	3673	MK328035	Yes	5	Tuva Republic, Kurtushibinsky Range	51.975	93.965	Bodrov et al. (2020)
–	3486	MK328036	–	6	Buryatia Republic, East Sayan Range	52.347	100.752	Bodrov et al. (2020)
–	3495	MK328037	–	6	Buryatia Republic, East Sayan Range	52.347	100.752	Bodrov et al. (2020)
–	3497	MK328038	–	6	Buryatia Republic, East Sayan Range	52.347	100.752	Bodrov et al. (2020)
–	3467	MK328035	Yes	6	Buryatia Republic, East Sayan Range	52.347	100.752	Bodrov et al. (2020)
–	25	KC962259	Yes	7	Kazakhstan, Hamir River	50.195	84.585	Bodrov et al. (2020)
*A. lemminus*	3738	MK328040	Yes	8	Yakutia Republic, Kurong‐Honku River	57.509	126.547	Bodrov et al. (2020)
–	4854	OQ992607	–	9	Yakutia Republic, Kuranakh Dyby pass	62.801	138.934	SAMN35742104
–	4853	MK341107	Yes	9	Yakutia Republic, Kuranakh Dyby pass	62.801	138.934	Bodrov et al. (2020)
–	3741	MK328041	Yes	10	Yakutia Republic, Barylas River	56.972	125.391	Bodrov et al. (2020)
–	3739	MK328042	–	10	Yakutia Republic, Barylas River	56.972	125.391	Bodrov et al. (2020)
–	4669	MK341116	–	11	Yakutia Republic, Mount Evota	57.541	125.136	Bodrov et al. (2020)
–	4668	MK341115	–	11	Yakutia Republic, Mount Evota	57.541	125.136	Bodrov et al. (2020)
–	3894	MK341103	Yes	12	Buryatia Republic, Severo‐Baykalsky District	55.709	109.073	Bodrov et al. (2020)
–	3939	MK341104	Yes	13	Buryatia Republic, Muysky District	56.327	113.214	Bodrov et al. (2020)
–	5013	OQ992612	–	14	Yakutia Republic, Tiksi	71.614	128.681	SAMN35742099
–	5012	OR651410	Yes	14	Yakutia Republic, Tiksi	71.614	128.681	Current study
–	5010	OR651411	Yes	14	Yakutia Republic, Tiksi	71.614	128.681	Current study
–	5018	OQ992615	–	15	Yakutia Republic, Nelkansky pass	64.568	143.232	SAMN31394358
–	5019	OQ992616	–	15	Yakutia Republic, Nelkansky pass	64.568	143.232	SAMN31394359
–	5017	OQ992614	–	15	Yakutia Republic, Nelkansky pass	64.568	143.232	SAMN31394357
–	4856	MK341108	Yes	15	Yakutia Republic, Nelkansky pass	64.568	143.232	Bodrov et al. (2020)
–	564	MK341114	–	16	Chukotka Autonomous area, Keveem River	69.354	173.669	Bodrov et al. (2020)
–	555	MK328039	–	17	Chukotka Autonomous area, Anadyr River	66.808	169.124	Bodrov et al. (2020)
–	554	OQ992613	Yes	17	Chukotka Autonomous area, Anadyr River	66.808	169.124	SAMN13341606
–	–	KJ556632	–	17	Chukotka Autonomous area, Anadyr River	66.766	169.566	Kohli et al. (2014)
–	–	KJ556633	–	17	Chukotka Autonomous area, Anadyr River	66.766	169.566	Kohli et al. (2014)
–	–	KJ556634	–	17	Chukotka Autonomous area, Anadyr River	66.766	169.566	Kohli et al. (2014)
–	–	KJ556635	–	17	Chukotka Autonomous area, Anadyr River	66.766	169.566	Kohli et al. (2014)
–	–	KJ556621	–	18	Republic of Sakha, Stokovo station	61.847	147.662	Kohli et al. (2014)
–	4445	OQ992611	Yes	19	Khabarovsk Krai, Bureinskiy Range	51.3	134.331	SAMN35742103
*A. tuvinicus*	5724	–	Yes	20	Republic of Khakassia, Shirinsky District	54.469	90.198	–
*–*	2472	KC962271	Yes	21	Tuva Republic, Piy‐Khemsky District	52.055	94.355	Bodrov et al. (2020)
A. semicanus	5752	–	Yes	22	Tuva Republic, Piy‐Khemsky District	50.561	94.861	–
*A. olchonensis*	3124	–	Yes	23	Irkutsk Oblast, Olkhon	53.063	106.987	–
–	3125	KC962261	Yes	23	Irkutsk Oblast, Olkhon	53.063	106.987	Bodrov et al. (2020)

*Note*: Tissue IDs in the tissue collection of Zoological Institute RAS, and GenBank accession numbers for *cytb* sequences used in this study. QuaddRAD‐seq column indicates the samples for which the quaddRAD‐seq data were obtained. Site ID refers to the sample sites in Figure 1.

To examine phylogenetic patterns in the nuclear genome, we used the quaddRAD‐seq (Franchini et al., 2017) dataset, which consisted of 20 DNA samples from representatives of the genus *Alticola*. Within the subgenus *Alticola*, three species were selected: *A. tuvinicus*, *A. semicanus*, and *A. olchonensis* (five DNA samples were sequenced) as an outgroup. Of the 15 *Aschizomys* DNA samples, five belong to *A. macrotis*, five come from *A. lemminus* (the group that carries a mitochondrial genome similar to that of *A. macrotis*; hereafter, we refer to this group as the *A. lemminus* western lineage, marked in green in Figure 1), and the last five DNA samples belong to specimens from all other *A. lemminus* sampling sites (Table 1).

All new tissue samples used in this study were deposited in the tissue collection of the Evolutionary Genomics and Paleogenomics Laboratory (the Zoological Institute of the Russian Academy of Sciences [ZIN RAS]).

### 2.1 cytb dataset preprocessing and tree calibration

To estimate the divergence time of *Aschizomys* species' populations, we examined the *cytb* dataset. To obtain new *cytb* sequences from genome‐wide sequencing data from previous studies (Abramson et al., 2021; Bondareva et al., 2023), we aligned the raw reads to a reference *cytb* sequence in BWA‐MEM aligner (Li, 2013). SAM files were then sorted using SAMtools (Li et al., 2009). Only mapped reads were retained, and the SAM files were converted into bam files. In the following analysis, a consensus *cytb* sequence for each DNA sample was used. Next, all *cytb* sequences were aligned by the MAFFT algorithm (Katoh et al., 2019) and manually trimmed.

To assess times of divergence of major clades in the subgenus, we chose a secondary calibration approach (the one that involves available information on the sequence mutation rate). By means of the clock rate previously reported for *Microtus agrestis* (Herman et al., 2014), estimated at 4.572 × 10^−7^ substitutions/(site·year) (95% highest posterior density interval: 3.411–5.834 × 10^−7^ substitutions per site per year), we calibrated our tree in Beast2 (Drummond & Rambaut, 2007). As in ref. (Herman et al., 2014), these demographic analyses were performed via a two partition model (first and second codon positions linked; the third codon position separate). We chose the HKY site model as recommended for protein‐coding nucleotide data (Shapiro et al., 2006), with a gamma distribution of rates between sites, and checked the estimation box for the substitution rate for each partition, allowing their substitution rate to differ from the clock rate we used as a prior. A strict clock model was employed, and uncertainty in the prior clock rate was set up as a normal distribution in the prior tab (mean = 4.572 × 10^−7^, sigma = 5 × 10^−5^). We also tried our analyses with the UCLN clock model. The two potentially appropriate tree priors—the coalescent constant population size model and the skyline demographic model—were tested and compared. Posterior distributions of these and other model parameters were derived from four or more independent Markov chain Monte Karlo simulations, each run for 100‐million generations, until the effective sample size for each parameter was sufficient (200 or more).

The clock model had no significant effect on the estimated divergence time, and therefore here we report results on a strict clock model. When the Bayesian skyline was used as the tree prior, divergence times were slightly greater as compared to the coalescent constant population size prior. To choose between these two priors, model selection with the nested sampling approach (Bouckaert et al., 2019) was applied, and there was strong support for the coalescent constant population size model (marginal likelihood is −2809 for this model and −2900 for the skyline model). The results obtained with the strict clock model and the coalescent constant population size tree model are reported here.

### 2.2 DNA extraction and quaddRAD‐seq library preparation

Total DNA was isolated from muscle tissue samples with the QIAamp DNA Mini Kit (lot # 160049272) and eluted with 50 μL of nuclease‐free water. Concentration of the purified DNA was measured on a Qubit 2.0 fluorometer.

The quaddRAD‐seq library was prepared according to a standard protocol (Franchini et al., 2017). For restriction digestion and ligation of adapters, a mixture consisting of 6 μL of 10× CutSmart Buffer (cat. # B6004S, New England Biolabs) and 0.4 μL of PstI‐HF (20 U/μL) (cat. # R0140S, New England Biolabs) was added to 600 ng of purified DNA, 0.4 μL of MspI (U/μL) (cat. # R0106S, New England Biolabs), 0.6 μL of T4 ligase (400 U/μL) (cat. # M0202S, New England Biolabs), 6 μL of 10 mM ATP (cat. # P0756S, New England Biolabs), 0.7 μL of the quaddRAD‐i5 adapter, 0.7 μL of the quaddRAD‐i7 adapter, and nuclease‐free water to a total volume of 60 μL. The reaction mixture was incubated for 3 h at 30°C (on a T100 Thermal Cycler; Bio‐Rad). Thereafter, the resulting fragments were purified and “sized” using magnetic particles (AmpureXP beads, Beckman Coulter, cat. # A63882). For this purpose, 0.4 volumes of magnetic particles was added to each sample. The supernatant was incubated for 10 min with intermittent stirring and transferred to a magnetic stand; the supernatant was transferred into clean test tubes, 0.4 volumes (relative to the initial sample volume) of magnetic particles was added to it, and the supernatant was incubated for 5 min with intermittent stirring. The tubes were placed on a magnetic rack, the supernatant was discarded, and the magnetic particles with bound DNA were washed two times with 200 μL of 80% ethanol. After the second washing, the alcohol was carefully removed, the magnetic particles were dried at room temperature for ~2 min, and 20 μL of 0.1 TE buffer was added for elution and incubated 2 min on the table at room temperature. Next, the tubes were placed on the magnetic rack, and the supernatant (containing the desired DNA fragments) was transferred into new test tubes. The concentration of the resulting fragments with adapters was measured on the Qubit 2.0 fluorometer.

Index PCR was carried out as follows: to 50 ng of DNA (after restriction digestion, ligation, and purification), a mixture was added consisting of 20 μL of 5× Phusion HF Buffer, 0.5 μL of Phusion high‐fidelity DNA polymerase (Phusion® HF DNA polymerase; 2 U/μL, cat. # M0530 S, New England Biolabs), 2 μL of dNTPs (10 mM each) (cat. # N0447S, New England Biolabs), 4 μL each of 10 μM PCR primer quaddRAD‐i5n and PCR primer quaddRAD‐i7n, and nuclease‐free water to a total volume of 100 μL. PCR (15 cycles) was conducted on the T100 Thermal Cycler (Bio‐Rad) under the following conditions: 98°C for 30 s; 15 cycles of 98°C for 10 s, 65°C for 30 s, and 72°C for 30 s; followed by 72°C for 5 min. PCR products were purified on magnetic beads (0.8× volume) (AmpureXP beads Beckman Coulter, cat. # A63882) and eluted with 20 μL of nuclease‐free water. The concentration of the obtained libraries was measured on the Qubit 2.0 fluorometer. The quality and length of the libraries were checked by means of an Agilent 2100 Bioanalyzer automated electrophoresis system.

### 2.3 QuaddRAD‐seq data processing

Sequence reads from the DNA samples were demultiplexed by means of outer indexes in bcl2fastq 2.20 (https://support.illumina.com/downloads/bcl2fastq‐conversion‐software‐v2‐20.html). Then, to remove adapters and perform quality filtering, the reads were processed with Fastp (Chen et al., 2018) with default settings. Thereafter, PCR duplicates were removed using the clone_filter program from the Stacks 2.6.0 software suite (Catchen et al., 2013). Thereafter, the data from the DNA samples were demultiplexed by means of inner indexes using the process_radtags program from Stacks 2.6.0.

The output reads from the Stacks package were aligned to the *Microtus ochrogaster* reference genome (GCA_000317375.1) in BWA‐MEM. After obtaining *.SAM files, we compressed them to *.bam format and sorted and indexed the alignments using SAMtools v.1.14 (Li et al., 2009).

To find loci orthologous between samples and make a SNP call, we utilized the ref_map.pl script implemented in Stacks (v.2.5) (Catchen et al., 2013). We selected the following settings for the ref_map.pl script: ‐p 4 (a locus must be present in all four populations to be written in the vcf file), −r 0.6 (at least 60% of individuals in a population must have the information in a locus to process the locus), and ‐write‐single‐snp (only the first SNP per locus was recorded). The latter setting was chosen to reduce effects of linkage disequilibrium in subsequent analyses involving Patterson's *D* test (Durand et al., 2011).

### 2.4 Phylogenetic analysis of the quaddRAD‐seq data

At the first step of the analysis, we evaluated phylogenetic relationships between all quaddRAD‐seq samples on the basis of the total number of SNPs. For this analysis, we converted our vcf file to the nexus format, leaving only those SNPs for which all samples had information. Then, we ran MrBayes v.3.2.7a (Ronquist & Huelsenbeck, 2003) with the GTR + I nucleotide substitution model, four Markov chain Monte Karlo (three heated chains and one cold chain), and 500,000 generations. All other settings were left at default values. We chose a nucleotide substitution model in the MEGA software (Tamura et al., 2021).

To investigate genetic structure within the *Aschizomys* subgenus, we kept only DNA samples from this subgenus in our SNP dataset and performed principal component analysis (PCA) by means of the R package adegenet (Jombart, 2008) with the glPca command.

### 2.5 A test for introgression

We evaluated a hypothesis of genetic introgression between *A. macrotis* and *A. lemminus* western populations by the classic ABBA‐BABA test in our SNP dataset. Briefly, this test counts two variants of discordant SNPs (ABBA and BABA) that give a topology different from that of the main signal in the SNP array. In our case, the main signal suggested that all *A. lemminus* samples represent a monophyletic clade sister to *A. macrotis*. Both discordant SNP variants can be caused by ILS (Pamilo & Nei, 1988) under the null hypothesis of no introgression. Nevertheless, the occurrence of genetic introgression between some nonsister lineages (P2 and P3 in Figure 5) may result in prevalence of ABBA‐like SNPs over BABA‐like SNPs; this outcome can be assessed with a single statistic, D (Green et al., 2010).

The *D* test was performed using the Dsuite (Malinsky et al., 2021) tool with default settings. Five specimens of the subgenus *Alticola* served as an outgroup and all five samples of *A. macrotis* as lineage P3 (Figure 4; population from which we supposed the introgression proceeded). All samples of the so‐called western lineage of *A. lemminus* were pooled and designated as population P2, while all other *A. lemminus* samples as population P1. Then, we performed the *D* test at the individual level by comparing all the pairs of the *A. lemminus* samples individually (Table 3).

## 3 RESULTS

### 3.1 A cytb phylogenetic pattern and divergence time estimation

A total of 40 *cytb* sequences were analyzed. The length of the alignment was 1143 bp, with 166 variable sites, of which 129 were parsimony‐informative sites. Pairwise nucleotide distances between *A. macrotis* and *A. lemminus* populations are given in Table 2.

**TABLE 2:** *cytb p*‐distances between populations of the subgenus *Aschizomys.*

	A. macrotis	*A. lemminus* western	*A. lemminus* Bureya	*A. lemminus* North Yakutia
*A. macrotis*				
*A. lemminus* western	0.0273			
*A. lemminus* Bureya	0.0662	0.0590		
*A. lemminus* North Yakutia	0.0703	0.0613	0.0358	
*A. lemminus* Chukotka	0.0686	0.0602	0.0364	0.0167

**FIGURE 2:**
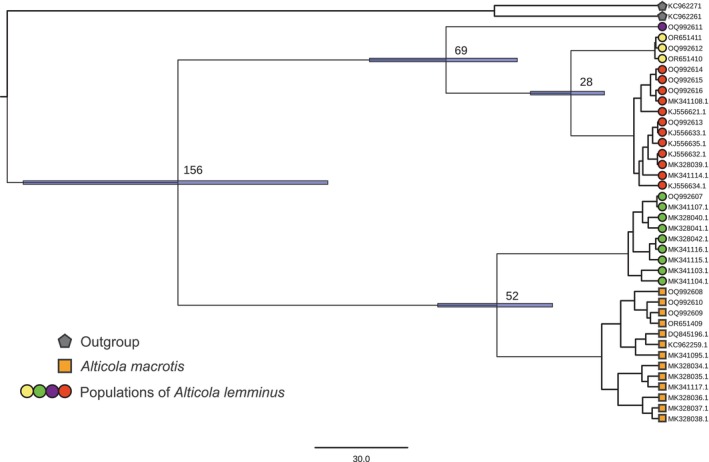
The phylogenetic tree reconstructed from *cytb* gene sequences in Beast2. All tree nodes have Bayesian posterior probabilities >0.95. Numbers near the nodes are mean divergence times in thousands of years before present, and the blue bars are 95% credible intervals. The numbers at the tips denote the Genbank ID presented in Table 1. Color codes are the same as in Figure 1.

The Bayesian *cytb* tree (Figure 2) shows the same pattern as reported previously (Bodrov et al., 2020). The main split within the subgenus *Aschizomys* divides all samples into two sister clades, one containing only *A. lemminus* specimens, and the other *A. macrotis* and specimens from the “western” lineage of *A. lemminus*. The time of divergence between these two clades was estimated to be 156 thousand years before present (thousand years ago) (95% credible interval: 107–207 thousand years ago). The split between *A. macrotis* and the western population of *A. lemminus* took place ~52 thousand years ago (95% credible interval: 35–72 thousand years ago).

### 3.2 Phylogenetic analysis of the quaddRAD‐seq data

**FIGURE 3:**
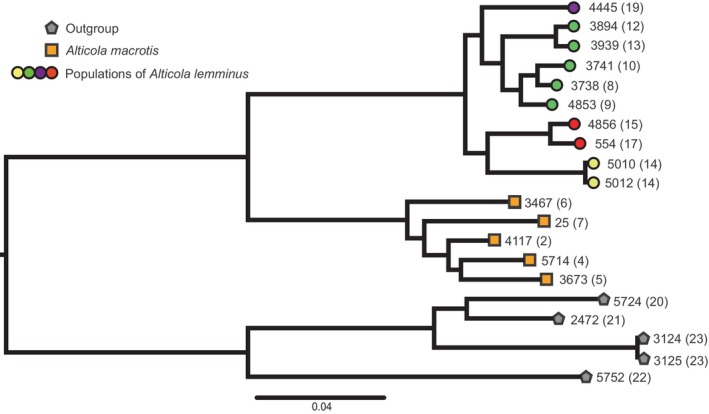
The tree generated by MrBayes from the quaddRAD‐seq data. All tree nodes have Bayes posterior probabilities >0.95. The numbers at the tips correspond to the tissue ID in Table 1, numbers in brackets correspond to the site ID in Figure 1. Color labeling is the same as in Figure 1. The gray tips denote samples from the subgenus *Alticola* serving as an outgroup.

The mean coverage per sample, recorded in the gstacks log, was 7.7×, with SD = 1.8. The final nexus matrix that we constructed from the vcf file contained 27,309 SNPs for 20 individuals. This dataset yielded a tree very similar to those for *BRCA* and *LCAT* loci from our previous study (Bodrov et al., 2020), where *A. macrotis* and *A. lemminus* appeared to be monophyletic species. *A. lemminus* samples were next distributed into two groups, with individuals from the Bureya mountain range and western samples in one group and individuals from North Yakutia and Chukotka in the other (Figure 3).

**FIGURE 4:**
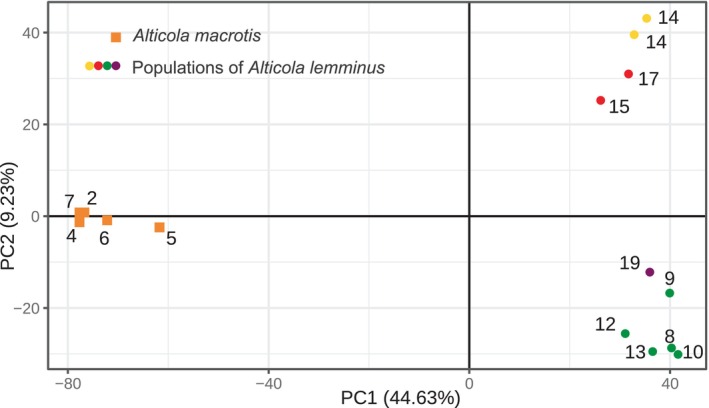
Subdivision of *Aschizomys* DNA samples by principal component analysis (PCA). The numbers next to the point correspond to sampling sites' ID numbers in Figure 1. Color codes are the same as in Figure 1.

PCA was performed only on DNA samples of the subgenus *Aschizomys* in the quaddRAD‐seq dataset. The vcf file contained 122,199 variants. The bulk of genetic variance (44.63%, Figure 4) is explained by principal component 1 (PC1), and all our *Aschizomys* DNA samples showed strong divergence into two groups along PC1: one group containing *A. macrotis* individuals and the other group comprising *A. lemminus* individuals. While *A. macrotis* individuals formed a dense population cluster, *A. lemminus* individuals separated into two clusters along PC2. These clusters match the first divergence of *A. lemminus* on the phylogenetic tree (Figure 3). One of them contains individuals from North and East Yakutia and Chukotka, and the second one unites individuals from the “western” lineage and the Bureya mountain range.

### 3.3 *D*‐statistic tests for introgression

**FIGURE 5:**
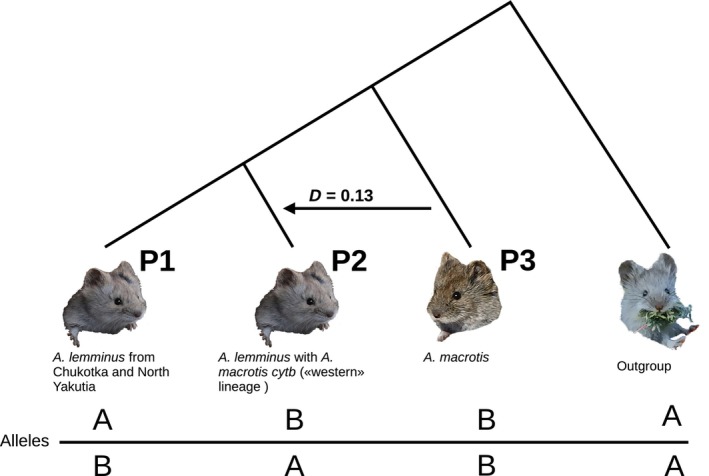
The four‐taxon ABBA‐BABA test scheme of our analysis. The branches are labeled as in Table 3. Contrary to the real topology, ABBA and BABA SNP patterns occur in equal proportions in the genome under ILS. The letters A and B denote the ancestral and derived states of the alleles, respectively. Post‐divergence gene flow (introgression) from lineage P3 to P2 generates additional instances of the ABBA pattern. The arrow indicates the direction of the introgression, and the value above the arrow is the result of Patterson's *D* test in population‐level analysis.

The resulting vcf file contained 122,199 SNPs that passed all filters. Patterson's *D* test performed on these data revealed significant genetic introgression between the “western” lineage of *A. lemminus* and *A. macrotis* at the population level (Table 3, first row; Figure 5). Individual‐level tests yielded similar results in all combinations where samples from the “western” lineage were compared with other samples of *A. lemminus* (Table 3, other rows).

**TABLE 3:** Results of the test of introgression from *A. macrotis* to the western population of *A. lemminus* relative to other *A. lemminus* individuals.

P1	P2	P3	*D*‐statistic	*p* value	BBAA	ABBA	BABA
*A. lemminus* ID (non‐“western”)	*A. lemminus* ID (“western”)	*A. macrotis*	0.130299	7.98E−11	8552.26	482.011	370.88
19	8	*A. macrotis*	0.083987	.0342	7477.47	327.345	276.62
15	8	*A. macrotis*	0.13786	1.50E−05	6049.18	373.895	283.295
14 (5010)	8	*A. macrotis*	0.074499	.01497	7398.45	364.63	314.067
14 (5012)	8	*A. macrotis*	0.11683	7.78E−05	6926.76	374.868	296.439
17	8	*A. macrotis*	0.070897	.03256	7077.58	391.345	339.528
19	10	*A. macrotis*	0.094169	.00852	7619.75	338.263	280.038
15	10	*A. macrotis*	0.139157	3.45E−05	6096.81	374.719	283.169
14 (5010)	10	*A. macrotis*	0.10194	.0034	7500.02	363.971	296.629
14 (5012)	10	*A. macrotis*	0.140676	3.82E−06	7053.83	378.128	284.861
17	10	*A. macrotis*	0.079043	.01658	7154.31	383.544	327.353
19	12	*A. macrotis*	0.170795	2.60E−05	5941.54	356.801	252.701
15	12	*A. macrotis*	0.200689	3.98E−12	4844.37	349.547	232.697
14 (5010)	12	*A. macrotis*	0.160228	2.65E−09	5988.2	351.616	254.499
14 (5012)	12	*A. macrotis*	0.193436	5.29E−11	5898.5	370.231	250.215
17	12	*A. macrotis*	0.143366	1.52E−06	5712.47	371.178	278.094
19	13	*A. macrotis*	0.184764	6.31E−06	6856.78	403.455	277.617
15	13	*A. macrotis*	0.198009	5.52E−12	5616.85	410.335	274.693
14 (5010)	13	*A. macrotis*	0.174205	4.22E−08	6906.02	403.144	283.523
14 (5012)	13	*A. macrotis*	0.215915	9.27E−11	6531.05	419.343	270.414
17	13	*A. macrotis*	0.153588	1.44E−09	6579.03	437.182	320.77
19	9	*A. macrotis*	0.082182	.00927	7333.66	316.199	268.174
15	9	*A. macrotis*	0.160853	1.37E−08	6328.14	320.112	231.4
14 (5010)	9	*A. macrotis*	0.08774	.03545	7482.85	337.44	283.002
14 (5012)	9	*A. macrotis*	0.139444	9.05E−05	7155.46	355.624	268.583
17	9	*A. macrotis*	0.106878	4.38E−05	7449.71	334.728	270.087

*Note*: The first row contains the *D*‐statistic, *p*‐value, and number of different SNP patterns (Figure 4) for the population‐level test. Other rows present these statistics for all pairwise combinations of *A. lemminus* DNA samples from the “western” lineage (P2) with all other *A. lemminus* DNA samples (P1). All individuals (in columns P1 and P2) are identified by their sampling site ID given in Figure 1.

## 4 DISCUSSION

The rock vole, genus *Alticola*, remains one of insufficiently studied genera of voles owing to its hard‐to‐access habitats, low abundance, and phylogenetic structure complicated by the cases of mito‐nuclear discordance described above. In this work, we focused on one taxon of rock voles: the subgenus *Aschizomys*. We present here the first genome‐wide study on this group; this paper aimed at understanding the causes of the mito‐nuclear discordance and at reconstructing the most likely evolutionary history, phylogeny, and taxonomy of the subgenus. We hypothesized that if the species in question represent distinct evolutionary lineages, then nuclear‐DNA data will not conflict with the conventional taxonomic division of the group, and hence the cause of the mito‐nuclear discordance may be introgression or ILS.

### 4.1 Mitochondrial phylogeny, the test for introgression, and divergence time estimation

Introgression and ILS result in different predictions regarding branch lengths of the gene trees that evolve in accordance with the underlying species phylogeny. Going back to the aforementioned mito‐nuclear discordance, if it is explained by ILS, then the *cytb* sequence of the *A. lemminus* “western” lineage and *A. macrotis* will have coalesced before the actual divergence of the two species (looking forward in time), whereas under introgression, by definition, this coalescence must have occurred after the divergence of the species. Therefore, under ILS, the time of the speciation event represents a lower bound of the minimum divergence time between these sequences (Joly et al., 2009). In the absence of deep coalescence of the *cytb* lineages, the estimated time of the first divergence in the *cytb* tree (Figure 2) can be assumed to be the time of the speciation event (156 thousand years ago, 95% credible interval: 107–207 thousand years ago). Our estimated time of divergence between *A. macrotis* sequences and the western lineage of *A. lemminus* is 52 ky BP (95% credible interval: 35–72 thousand years ago), which is considerably less than the estimated time of divergence of the species. The confidence intervals of the two divergence estimates do not overlap. In summary, we can reject the hypothesis that ILS leads to nonmonophyly of *A. lemminus*. Taking into account the confirmed monophyly of the two species in our nuclear‐DNA data, the mitochondrial introgression hypothesis can be accepted, and the time of *cytb* divergence between *A. macrotis* and the *A. lemminus* “western” lineage is likely to be the introgression time. Given that only one lineage of *A. lemminus* carries the *cytb* sequence that is the characteristic of all *A. macrotis* individuals being analyzed, it is logical to assume that *A. macrotis* is the donor of this *cytb* sequence.

Further examination of the tree indicates that the main clade of *A. lemminus* split into three other groups around the time of the presumed introgression (95% credible intervals overlap). It is therefore reasonable to suppose that the *A. lemminus* species was actively dispersing at that time. It has been shown in modeling papers (Currat et al., 2008) that under migration conditions, introgression is expected to proceed from an aboriginal species (*A. macrotis* in this case) to a dispersing one. It is also worth noting that the *A. lemminus* population with introgressed *cytb* is a sister clade to all *A. macrotis* individuals under study, and no shared or similar haplotypes between these two clades were detected. It is therefore likely that the introgression proceeded from an extinct *A. macrotis* population or from the one that has not been recorded yet. Otherwise, one could expect the *A. lemminus* lineage with the introgressed *cytb* to be closer to one of the recent lineages of *A. macrotis* (the source of hybridization) than to another lineage.

The secondary calibration method was recently actively criticized (Schenk, 2016); however, in the absence of a good fossil record, the method can be used as suitable for rough estimation of timing of major divergence splits. We applied this approach to assess the time of divergence within *Aschizomys cytb* lineages. Our estimate of the time of divergence between *A. lemminus* and *A. macrotis* differs significantly from that made previously (Abramson et al., 2021; Bodrov et al., 2020), which was obtained via fossil calibration and was estimated to be within 1–2 million years ago. This discrepancy in speciation time estimates is a direct consequence of the difference in the techniques utilized for tree calibration. It has been recognized for some time that the estimated rate of change (substitution or mutation rate) depends on the order of magnitude of the age of the calibration point (Ho et al., 2005). The rate of change used here was derived from biogeographic calibration close to 10,000 years old (Herman et al., 2014). The calibration point employed in the previous studies is the divergence of *Alticola* and *Clethrionomys* (Tesakov, 1996), which is approximately 2.6 million years ago. We believe that the calibration approach used here is more appropriate because smaller time intervals are considered. Nonetheless, further research on ancient sequences is needed to make more accurate time estimates of species and population divergence.

### 4.2 Nuclear phylogeny and admixture distribution

We present the first genome‐wide nuclear sequences (quaddRAD‐seq) for members of the genus *Alticola*. Contrary to the *cytb* tree (Figure 2), the tree constructed from these data (Figure 4) points to the monophyly of *A. lemminus*. The species *A. lemminus* itself is divided into four separate lineages corresponding to the lineages on the *cytb* tree, but in contrast to the *cytb* tree, individuals from the “western” lineage constitute a sister clade to the lineage from the Bureya mountain range.

Phylogenetic‐tree construction and PCA are techniques that extract the main phylogenetic signal from an input dataset. In other words, they represent the primary history, that is, the “central trend in the rich patchwork of evolutionary history” (Wolf et al., 2002). At the same time, the quaddRAD‐seq approach that we applied allows for an analysis of thousands of loci throughout the nuclear genome. Some of these loci may carry a signal of minor history, such as those that would arise for example due to either introgression or ILS (Baum, 2007). In the mitochondrial genome of the *Aschizomys* subgenus, we obviously see a trace of such minor history, which manifests itself as the mito‐nuclear discordance. We tried to find traces of it in the nuclear genome by the *D* test, which analyzes a distribution of the SNPs that do not match the main signal in the data.

Our *D*‐statistic results uncovered a high correlation between mitochondrial and nuclear phylogeographic patterns (Table 3; Figure 5). All DNA samples from the “western” *A. lemminus* lineage (DNA samples clustered with *A. macrotis* on the *cytb* tree) carried a signal of nuclear introgression from *A. macrotis*. Moreover, this signal (the proportion of ABBA sites) is much weaker than the main signal in the nuclear genome (the proportion of BBAA sites), which points to the monophyly of *A. lemminus*. That is why there are no signs of introgression in the nuclear tree.

It should be noted that the significant value of Patterson's *D* test does not necessarily mean introgression between the tested populations (Durand et al., 2011). There can be some other population history leading to it, such as certain ancient population structure or introgression from close but unanalyzed taxa. As stated above, the observed signs of introgressed *cytb* testifies in favor of gene flow from the ancient *A. macrotis* population. Because we see the geographic correlation between introgressed mitochondrial and nuclear genomes' patterns, we believe that it is all a consequence of one process in the past.

### 4.3 Possible drivers of the observed introgression in *Aschizomys* species

Many cases of unidirectional introgression of mitochondrial DNA owing to interspecies hybridization events have been described earlier (see Mallet, 2005 for review). Among mammals, probably one of the most well‐known cases is the case of the Arctic bear, the entire recent population of which has the mitochondrial genome of the Brown bear (Cahill et al., 2015; Edwards et al., 2011; Miller et al., 2012). Quite frequent cases of unidirectional introgression occur in rodents, including, in particular voles. Thus, within the tribe Clethrionomyini, to which the taxon under study belongs, the most well‐studied case is the introgression of mitochondrial genome of the *Clethrionomys rutilus* Pallas, 1779 to the *Clethrionomys glareolus* Schreiber, 1780, which has become a model system for studying end glacial colonization routes and introgression (Abramson et al., 2009; Filipi et al., 2015; Marková et al., 2020). Shifts in species distribution after the last glacial maximum in couple with differential demography of local and colonizing populations are considered to be among the main causes of unidirectional introgression and provide a strong support for the idea that environmental change triggers hybridization (Aguillon et al., 2022; Mastrantonio et al., 2016). The abovementioned case of *Clethrionomys* species convincingly illustrates this viewpoint. Rapid environmental change leading to habitat destruction of a mitochondrial DNA‐accepting species niche may also be among possible causes of mitochondrial introgression (Lehman et al., 1991).

In a previous study (Bodrov et al., 2020), via species distribution modeling, it was proposed that climatic changes during the Last Glacial Maximum were the main drivers of shifts of the *Aschizomys* species' geographic range. These range shifts resulted in secondary contact and hybridization between the two diverged species in the area south of Lake Baikal [see discussion in ref. (Bodrov et al., 2020)], and these processes may have been the cause of the introgression and of the observed mito‐nuclear discordance. In the current work, we convincingly demonstrated mitochondrial and nuclear introgression between *A. macrotis* and the “western” *A. lemminus* lineage, thereby supporting this hypothesis. Furthermore, the estimated divergence times of the *cytb* lineages confirm that the approximate period of *A. lemminus* species migration and mitochondrial introgression is within the Late Pleistocene.

From the nuclear phylogeny, it may be inferred that the ancestral *A. lemminus* population lived in the area of the Suntar‐Khayata mountain range because it lies between two major recent species lineages (Figure 1). We propose that *A. lemminus* spread south and north from this area thus colonizing the territories it now occupies. Quite possibly, during this colonization, the “western” lineage of *A. lemminus* may have come into secondary contact with the population of *A. macrotis*, and hybridization occurred. Subsequent backcrossing of hybrids with *A. lemminus* led to the observed introgression. Thus, it is one more example, showing the case of introgression when an expanding (*A. lemminus*) lineage overrides a static (*A. macrotis*) one, but is itself invaded by genes from the resident population due to sequential founder events during the spatial expansion (Edwards et al., 2016). However, in this case we did not find any recent hybrid “tension zone” as in example of *C. glareolus*/*C. rutilus* and no shared or similar haplotypes in the considered species. Analysis of ancient DNA from fossil remains may help to more precisely determine the timing of the hybridization events and localization of the supposed donor population of *A. macrotis*.

### 4.4 Taxonomic considerations

Our results of the phylogenetic analysis of genome‐wide data do not match the subspecific structure described elsewhere on the basis of morphological data for both *A. macrotis* and *A. lemminus*.

Three subspecies have been described within *A. macrotis*: *A. m*. *macrotis* Radde, 1862; *A. m. vinogradovi* Rasorenova, 1933; and *A. m. fetisovi* Galkina et Epifantseva, 1988. We do not have material from the latter subspecies, and thus the taxonomic evaluation of this isolated form is a matter of future investigations. At the same time, the material studied here covers the distribution of the two other subspecies, including their terra typica. As we can see in the phylogenetic reconstruction based on the genome‐wide data (Figures 3 and 4), genetic structure within *A. macrotis* is very shallow, and we cannot discern any independent clades corresponding to *A. m. macrotis* (individuals No. 1–5 and 7 in Figure 1) or *A. m. vinogradovi* (individual No. 6, Figure 1), thereby supporting their sister relationships.

*Alticola lemminus* was described by Miller in 1898, and since then, the systematic position of this species has changed many times (Musser & Carleton, 2005). Many authors have assumed that it is a subspecies of the previous species (Gromov & Baranova, 1981; Gromov & Erbajeva, 1995; Gromov & Polyakov, 1997), whereas others (Musser & Carleton, 2005; Pavlinov & Rossolimo, 1987) believe that it is an independent species. The latter viewpoint is supported not only by the examination of morphological data but also by molecular evidence reported in the current study and previous ones (Bodrov et al., 2020). Moreover, significant morphological and chromosomal differences between tissue samples from isolated populations from the Chukotka Peninsula and Yakutia have been detected (Bykova et al., 1978), and variations in morphological cranial features indicate possible existence of three independent lineages on the Chukotka Peninsula and in North Yakutia and South Yakutia and that these taxa can be described as subspecies (Vasileva & Vasilev, 1992). Nevertheless, the only subspecies that has been described is *A. lemminus vicina* Portenko et al. (1963) (reported as *A. macrotis vicina*). The range of this subspecies was outlined as extending from the mouth of the Lena River to the Olekma region, with a terra typica within the latter. Our data clearly show that DNA samples from the Kharaulakh Mountains (site 14, Figure 1) do not cluster with DNA samples from South Yakutia and the Transbaikal region that Portenko et al. (1963) assigned to this subspecies, *A. l. vicina*, and therefore this taxon is paraphyletic. The molecular data presented here also do not support the opinion of Vasil'eva (1999) on three independent lineages that she distinguished, as our results clearly support the subdivision into two sister groups: one is formed by the DNA samples from the Chukotka Peninsula and North Yakutia (Kharaulakh Mountains, near the mouth of the Lena River), and the other by DNA samples from South Yakutia and the Bureya mountain range (green and purple circles in Figure 1). The presumed boundary between these groups goes along the Suntar‐Khayata mountain range. Whether these detected lineages deserve to be taxonomically separated into subspecies is a matter of future research, which should include expanded datasets and morphological analysis.

## AUTHOR CONTRIBUTIONS

**Ivan A. Dvoyashov:** Formal analysis (lead); investigation (equal); methodology (equal); software (lead); visualization (lead); writing – original draft (equal); writing – review and editing (equal). **Semyon Yu. Bodrov:** Data curation (equal); investigation (equal); methodology (equal); resources (equal); writing – original draft (equal); writing – review and editing (equal). **Elena S. Glagoleva:** Data curation (equal). **Nikolai V. Mamaev:** Investigation (supporting). **Natalia I. Abramson:** Conceptualization (lead); data curation (equal); funding acquisition (lead); investigation (equal); project administration (lead); supervision (lead); writing – original draft (equal); writing – review and editing (equal).

## Data Availability

A .vcf file containing SNP from the quaddRAD‐seq dataset used here has been deposited in GitHub and is available at: https://github.com/ZaTaxon/Aschizomys/blob/main/populations.snps.vcf.7z. New *cytb* sequences obtained in this study from available genome‐wide data have been deposited in GenBank. Their accession numbers are listed in Table 1.
